# Clinical and Genomic Analysis of Liver Abscess-Causing *Klebsiella pneumoniae* Identifies New Liver Abscess-Associated Virulence Genes

**DOI:** 10.3389/fcimb.2016.00165

**Published:** 2016-11-29

**Authors:** Meiping Ye, Jianfei Tu, Jianping Jiang, Yingmin Bi, Weibo You, Yanliang Zhang, Jianmin Ren, Taohui Zhu, Zhuo Cao, Zuochun Yu, Chuxiao Shao, Zhen Shen, Baixing Ding, Jinyi Yuan, Xu Zhao, Qinglan Guo, Xiaogang Xu, Jinwei Huang, Minggui Wang

**Affiliations:** ^1^Institute of Antibiotics, Huashan Hospital, Fudan UniversityShanghai, China; ^2^Fifth Affiliated Hospital of Wenzhou Medical UniversityLishui, China; ^3^Department of Bioinformatics, SJTU-Yale Joint Center for Biostatistics, Shanghai Jiaotong UniversityShanghai, China; ^4^Nanjing First Hospital, Nanjing Medical UniversityNanjing, China; ^5^Sixth Hospital of Wenzhou Medical UniversityLishui, China

**Keywords:** liver abscess, hypervirulent *Klebsiella pneumoniae*, virulence, plasmid, genome comparative analysis

## Abstract

Hypervirulent variants of *Klebsiella pneumoniae* (hvKp) that cause invasive community-acquired pyogenic liver abscess (PLA) have emerged globally. Little is known about the virulence determinants associated with hvKp, except for the virulence genes *rmpA/A2* and siderophores (*iroBCD/iucABCD*) carried by the pK2044-like large virulence plasmid. Here, we collected most recent clinical isolates of hvKp from PLA samples in China, and performed clinical, molecular, and genomic sequencing analyses. We found that 90.9% (40/44) of the pathogens causing PLA were *K. pneumoniae*. Among the 40 LA-Kp, K1 (62.5%), and K2 (17.5%) were the dominant serotypes, and ST23 (47.5%) was the major sequence type. S1-PFGE analyses demonstrated that although 77.5% (31/40) of the LA-Kp isolates harbored a single large virulence plasmid varied in size, 5 (12.5%) isolates had no plasmid and 4 (10%) had two or three plasmids. Whole genome sequencing and comparative analysis of 3 LA-Kp and 3 non-LA-Kp identified 133 genes present only in LA-Kp. Further, large scale screening of the 133 genes in 45 LA-Kp and 103 non-LA-Kp genome sequences from public databases identified 30 genes that were highly associated with LA-Kp, including *iroBCD, iucABCD* and *rmpA/A2* and 21 new genes. Then, these 21 new genes were analyzed in 40 LA-Kp and 86 non-LA-Kp clinical isolates collected in this study by PCR, showing that new genes were present 80–100% among LA-Kp isolates while 2–11% in *K. pneumoniae* isolates from sputum and urine. Several of the 21 genes have been proposed as virulence factors in other bacteria, such as the gene encoding SAM-dependent methyltransferase and *pagO* which protects bacteria from phagocytosis. Taken together, these genes are likely new virulence factors contributing to the hypervirulence phenotype of hvKp, and may deepen our understanding of virulence mechanism of hvKp.

## Introduction

*K. pneumoniae* is a common opportunistic pathogen responsible for nosocomial infections, such as pneumonia and urinary tract infections (Podschun and Ullmann, 1998). In mid-1980s and 1990, a new hypervirulent variant of *K. pneumoniae* (hvKp) causing invasive pyogenic liver abscess (PLA), was first described in Taiwan, and subsequently found worldwide, especially in Asia (Cheng and Lin, 1986; Siu et al., 2012; Shon et al., 2013). Different from “classic” *K. pneumoniae* (cKp), the new variants of *K. pneumoniae* exhibit enhanced virulence features. In addition to PLA, hvKp also cause other invasive diseases including abscesses at other sites (e.g., eyes, brain, prostate, and kidney), necrotizing fasciitis, and severe pneumonia with bacteremia (Hu et al., 1999; Saccente, 1999; Hyun et al., 2014; Kim et al., 2014). Although hvKp infection appears to occur often in diabetic patients, a particularly disconcerting problem is its ability to cause community-acquired, life-threatening infection among young and healthy individuals (Pomakova et al., 2012).

hvKp strains often form colonies with a hypermucoviscous phenotype, which can be defined semi-quantitatively by a positive “string test,” a method that has been widely used for identification of hvKp (Siu et al., 2012). However, not all hvKp strains are hypermucoviscous since non-hypermucoviscous hvKP have been reported (Tan et al., 2014; Qu et al., 2015). Serotyping and sequence typing of hvKp have been widely reported. Most hvKp have K1 or K2 capsular serotypes. Capsular with mucoid phenotype protects bacteria from phagocytosis and bactericidal effect of serum (Lin et al., 2004; Yeh et al., 2010). However, non-K1/K2 serotype hvKp strains were also reported and some K1/K2 strains can also be cKp strains (Mizuta et al., 1983; Yu et al., 2008; Brisse et al., 2009). Several sequence types (ST) have shown to be associated with hvKp. For example, ST23 is most associated with the K1 serotype, whereas ST86 and ST65 are often associated with K2 serotype (Chung et al., 2008; Siu et al., 2011; Lin et al., 2014). hvKp strains with other ST types were also reported (Merlet et al., 2012; Luo et al., 2014). Overall, it is difficult to define a hvKp strain solely based on colony phenotype, serotyping or sequence typing.

Several virulence factors have been identified to be associated with hvKp, including iron acquisition systems salmochelin (*iroBCDN*)/aerobactin (*iucABCDiutA*), and the regulator of mucoid phenotype A gene (*rmpA/A2*) (Hsieh et al., 2008; Hsu et al., 2011). Both salmochelin/aerobactin systems and *rmpA/A2* are found almost in all reported hvKp strains locating on a large plasmid (Struve et al., 2015). Salmochelin/aerobactin was shown to enhance the virulence of *K. pneumoniae* in a mouse model (Nassif and Sansonetti, 1986). The important role of RmpA in virulence has been demonstrated in animal model, as deletion of *rmpA* decreased virulence 1000-fold (Nassif et al., 1989). However, transforming of these factors into a plasmid cured hvKp strain could not fully restore the hypervirulent phenotype (Nassif et al., 1989), indicating that there are additional virulence factors yet-to-be identified.

The first complete genome sequence of hvKp was reported for NTUH-K2044 (ST23, K1 serotype), a most studied liver abscess-causing *K. pneumoniae* (LA-Kp) isolated from Taiwan (Wu et al., 2009). The NTUH-K2044 harbors a 224-Kb plasmid (pK2044) carrying the virulence genes *iroBCDN, iucABCDiutA*, and *rmpA/A2*. pK2044 is similar to pLVPK, another plasmid that was found in the hvKp strain CG43 and is required for *K. pneumoniae*-caused pyogenic liver abscess (KLA) (Chen et al., 2004). Recently Holt et al. analyzed genomic sequences among invasive and non-invasive *K. pneumoniae* strains, and found that the presence of *rmpA*/*A2* and the iron acquisition systems (*iroBCDN, iucABCDiutA*) are significantly associated with *K. pneumoniae* causing invasive human infection (Holt et al., 2015). Another recent genomic comparison analysis between clonal complex 23 (CC23) *K. pneumoniae* isolates (a group of hvKp strains) with non-CC23 *K. pneumoniae* strains found that all the CC23 strains harbored a pK2044-like plasmid encoding *iroBCDN, iucABCDiutA*, and *rmpA* (Struve et al., 2015). Overall, these genomic analyses support the notion that the presence of large plasmid in hvKp, and that plasmid-encoded *iroBCDN, iucABCDiutA*, and *rmpA/A2* virulence genes are tightly associated with hvKp strains. In addition, several gene clusters have been proposed to be associated with hvKp (Struve et al., 2015). Nevertheless, whether all hvKp strains contain a pK2044-like plasmid and whether other genes on the plasmids are conserved among hvKp strains remain unclear.

Since string test, serotyping and sequence typing cannot definitely define a *K. pneumoniae* isolate as a hvKp strain, we took the clinical features into account in addition to the microbiological phenotypes as recently proposed by Siu et al. (2012) and Shon et al. (2013). Given that liver abscess is the representative clinical syndrome of hvKp infection, in this study, we solely focused on the hvKp isolates cultured from most recent LA subcutaneous drainage, which provided us clearly defined hvKp strains. We then performed clinical, molecular and genomic studies. We sequenced two representative LA-Kp clinical isolates. To the best of our knowledge, this is the first report of LA-Kp genome sequences from mainland China. We discovered that LA-Kp has a diverse range of plasmids. We further identified 21 new genes associated with LA-Kp, which may deepen our understanding of virulence mechanism of hvKp in general.

## Materials and methods

### Ethics statement

The CT-guided LA subcutaneous drainage and sample collection were performed with written informed consent from LA patients during January 2014 to January 2016, and were approved by Fifth Affiliated Hospital of Wenzhou Medical University Ethics Committee and Nanjing First Hospital Ethics Committee. The operating procedures were conducted in accordance with the national guidelines in China (Li and He, 2001). Isolates from blood, sputum, and urine samples were collected as part of the routine clinical management of patients, according to the national guidelines in China (Shang et al., 2015). Therefore, informed consent was not sought.

### Clinical bacterial isolation

Besides LA-causing bacteria, clinical *K. pneumoniae* isolates collected from blood, sputum and urine samples were also included for comparative study of PCR screen of LA-associated genes. Blood samples were taken from patients who has bloodstream infections but without LA. Sputum and urine samples were taken from patients who had pneumonia or urinary tract infections, but without bacteremia. LA drainage, sputum, and urine samples were plated on Columbia blood agar plates and incubated at 37°C for bacterial isolation. Blood samples were first incubated in blood culture bottles and the presence of bacteria in blood culture was detected by BacT/ALERT system. Positive blood cultures were then plated on Columbia blood agar plates and incubated at 37°C for bacterial isolation. Strains of positive cultures were identified by VITEK 2 Compact System (bioMérieux, France). All isolates were stored in 25% (v/v) glycerol broth at minus 80°C until use.

### Antimicrobial susceptibility testing

Minimum inhibitory concentrations (MICs) of antimicrobial agents were determined by the agar dilution method and interpreted following the recommendations of the Clinical and Laboratory Standards Institute (CLSI, Wayne, PA, USA). *Escherichia coli* ATCC 25922 was used as the quality control strain.

### String test

The hypermucoviscous phenotype of *K. pneumoniae* was identified by a positive string test, which is defined as the formation of a viscous string >5 mm in length when a colony grown overnight on a sheep blood agar plate at 37°C was stretched by a bacteriology inoculation loop (Siu et al., 2012).

### Multilocus sequence typing (MLST) and serotyping of *K. pneumoniae*

MLST was performed by PCR amplification and subsequent sequencing of seven housekeeping genes of *K. pneumoniae* according to protocols provided on the MLST website for *K. pneumoniae* (http://bigsdb.pasteur.fr/klebsiella/klebsiella.html). Capsular serotype of each *K. pneumoniae* isolate was performed by PCR of the serotype-specific genes for K1, K2, K5, K20, K54, and K57 serotypes as previously reported (Turton et al., 2010).

### PCR detection of the targeted genes

Genomic DNA was extracted from all *K. pneumoniae* isolates using bacterial genomic DNA extraction kit (TIANGEN Biotech, Beijing, China). Targeted genes were amplified by polymerase chain reaction (PCR). PCR primers for each target genes are listed in Table S1.

### S1-pulsed-field gel electrophoresis (S1-PFGE) for detecting and sizing plasmids of LA-Kp

S1-PFGE was carried out for detection and determining the size of the endogenous plasmids in all clinical LA-Kp isolates as described previously (Barton et al., 1995). Briefly, Total DNA of LA-Kp were embedded in agarose gel plugs. The plugs were digested with S1 nuclease (TaKaRa) and then separated by PFGE. Genomic DNA of *Salmonella enterica* serovar Braenderup H9812 digested with XbaI was used as a molecular standard.

### Genome sequencing

Genomic DNA of clinical *K. pneumoniae* isolates GN-2, GN-3, and XL-1 were extracted using bacterial genomic DNA extraction kit, and sequenced using Illumina MiSeq sequencing technologies as described previously (Etienne et al., 2012). Fragment libraries were constructed using the Nextera kit (Illumina, San Diego, CA) followed by 300-bp paired-end sequencing on a MiSeq sequencer (Illumina) according to the manufacturer's instructions. The sequencing reads were assembled using SPAdes V3.5 with default parameters to include only contigs of more than 500 nucleotides (Bankevich et al., 2012). The genes were predicted and annotated using PATRIC online tool (Wattam et al., 2014).

### Genome comparative analysis

To identify highly conserved genes specifically present in LA-Kp, three LA-Kp genomes (GN-2, GN-3, and NTUH-K2044) and three non-LA-Kp genomes (XL-1, HS11286, and MGH78578) were compared using whole genome orthologous gene comparative analysis. To reduce bias introduced by different annotation platforms or parameters, the genomes of NTUH-K2044, HS11286, and MGH78578 were re-annotated by using PATRIC online tool. The protein sequences of the six *K. pneumoniae* strains were clustered into customized orthology clusters by orthoMCL (v1.4) with default parameters (Li et al., 2003). Genes in orthology clusters containing only genes from LA-Kp were considered as LA-associated gene. The LA-associated genes were identified by in-house scripts. BLASTN atlases of the chromosomes and plasmids of GN-2, GN-3, CG43, and NTUH-K2044 were constructed using BLAST Ring Image Generator (BRIG v0.95) with default parameters (Alikhan et al., 2011). The genome sequences of *K. pneumoniae* isolates from liver abscess (*n* = 44), blood (*n* = 45), and sputum and urine (*n* = 59) (Table S2) were downloaded from the European Nucleotide Archive (ENA; http://www.ebi.ac.uk/ena) (Bialek-Davenet et al., 2014; Holt et al., 2015; Struve et al., 2015). The genome sequences were screened for LA-specific genes using a read mapping approach with SRST2 (Inouye et al., 2014).

### Functional comparative analysis

Protein sequences of the three LA-Kp (GN-2, GN-3, and NTUH-K2044) and three non-LA-Kp (XL-1, HS11286, and MGH78578) were aligned to the Clusters of Orthologous Groups (COGs) (latest version) database via BLAST (v2.2.26) (*E* < 1e^−5^), and the best hits were selected as the COG annotations (Ye et al., 2006; Galperin et al., 2015). The COG clusters are referred to as the finished COG categories. The virulence factors were annotated by PATRIC online tool with PATRIC VF database. The presence of antibiotic resistance coding genes of the above six *K. pneumoniae* strains was investigated by using of the web-based tool ResFinder at http://cge.cbs.dtu.dk/services/ResFinder (Zankari et al., 2012).

### Nucleotide sequence accession numbers

The accession numbers for the three *K. pneumoniae* isolates sequenced in this study are available at DDBJ/ENA/GenBank under the bioproject PRJNA349219.

## Results

### Clinical characteristics of patients with *K. pneumoniae*-caused liver abscesses

A total of 68 patients diagnosed with liver abscess (LA) were enrolled from January 2014 to January 2016. Among them, 57 (83.8%) patients received percutaneous drainage therapy. Pus samples were collected and overall positive bacterial culture was 77.2% (44/57), of which, 90.9% (40/44) were identified as *K. pneumoniae* (Table 1). Other pathogens were *E. coli* (4.5%), *Klebsiella oxytoca* (2.2%), and *Candida albicans* (2.2%).

**Table 1:** **Culture results of liver abscess drainage samples from 57 patients**.

**Pathogens**	**Numbers of positive culture (%)**
Culture positive	44 (77.2)
*Klebsiella pneumoniae*	40 (90.9)
*Escherichia coli*	2 (4.5)
*Klebsiella oxytoca*	1 (2.2)
*Candida albicans*	1 (2.2)
Culture negative	13 (22.8)
Total samples	57

As shown in Table 2, all 40 patients with *K. pneumoniae*-caused liver abscesses (KLA) were community-acquired, and 55% (22/40) were diagnosed with underlying disease of diabetes. Most of KLA (67.5%) happened in the right lobe of liver. Eight patients (20.0%) had metastatic infections, including 4 patients with lung abscess, 3 with eye abscess and 1 with endocarditis. Blood leukocyte and C-reaction protein were elevated in 32 (80.0%) and 39 (97.5%) LA patients, respectively. Blood test showed disordered liver function in 35 KLA patients. None of the patients died after appropriate treatment.

**Table 2:** **Clinical characteristics of patients with *K. pneumoniae*-caused liver abscess**.

**Characteristics**	Values^*^
**GENDER**	
Male	21 (52.5%)
Female	19 (47.5%)
Average age	59.3 (33–81)
**TYPE OF ACQUISITION**	
Community-acquired	40 (100.0%)
Hospital-acquired	0
**EXISTING DISEASES**	
Diabetes mellitus	22 (55.0%)
Biliary tract diseases	8 (20.0%)
Urological stone/infection	4 (10.0%)
Biliary/gastrointestinal malignancies	2 (5.0%)
None	9 (22.5%)
Fever (Tmax>38.5 °C)	40 (100.0%)
**ABSCESS CHARACTER**	
Right lobe	27 (67.5%)
Left lobe	3 (7.5%)
Bilobar	10 (25.0%)
Gas in abscess	6 (15.0%)
**COMPLICATIONS**	
Lung abscess	4 (10.0%)
Eye abscess	3 (7.5%)
Endocarditis	1 (2.5%)
**INITIAL LAB EXAMINATION**	
White cell count (× 10^9^/L)	13.5 (4.3–23.3)
C-reaction protein (mg/L)	149.0 (5.2–312.0)
Serum albumin (g/L)	29.5 (19.3–60.1)
Alanine aminotransferase	69.0 (18.3–185.0)
Alkaline phosphatase (U/L)	169 (48.0–464.0)
Direct bilirubin (U/L)	10.3 (3.8–29.3)
Mortality	0

* *Data presented as No. (%) or mean (range)*.

### Microbiological characteristics of LA-Kp

All the 40 LA-Kp isolates were susceptible to the 10 antimicrobials tested including cefazolin, cefepime, cefotetan, aztreonam, levofloxacin, ciprofloxacin, amikacin, imipenem, piperacillin-tazobactam, and ampicillin-sulbactam. All LA-Kp isolates were resistant to ampicillin, and two isolates were resistant to SMZ-TMP.

**Figure 1:**
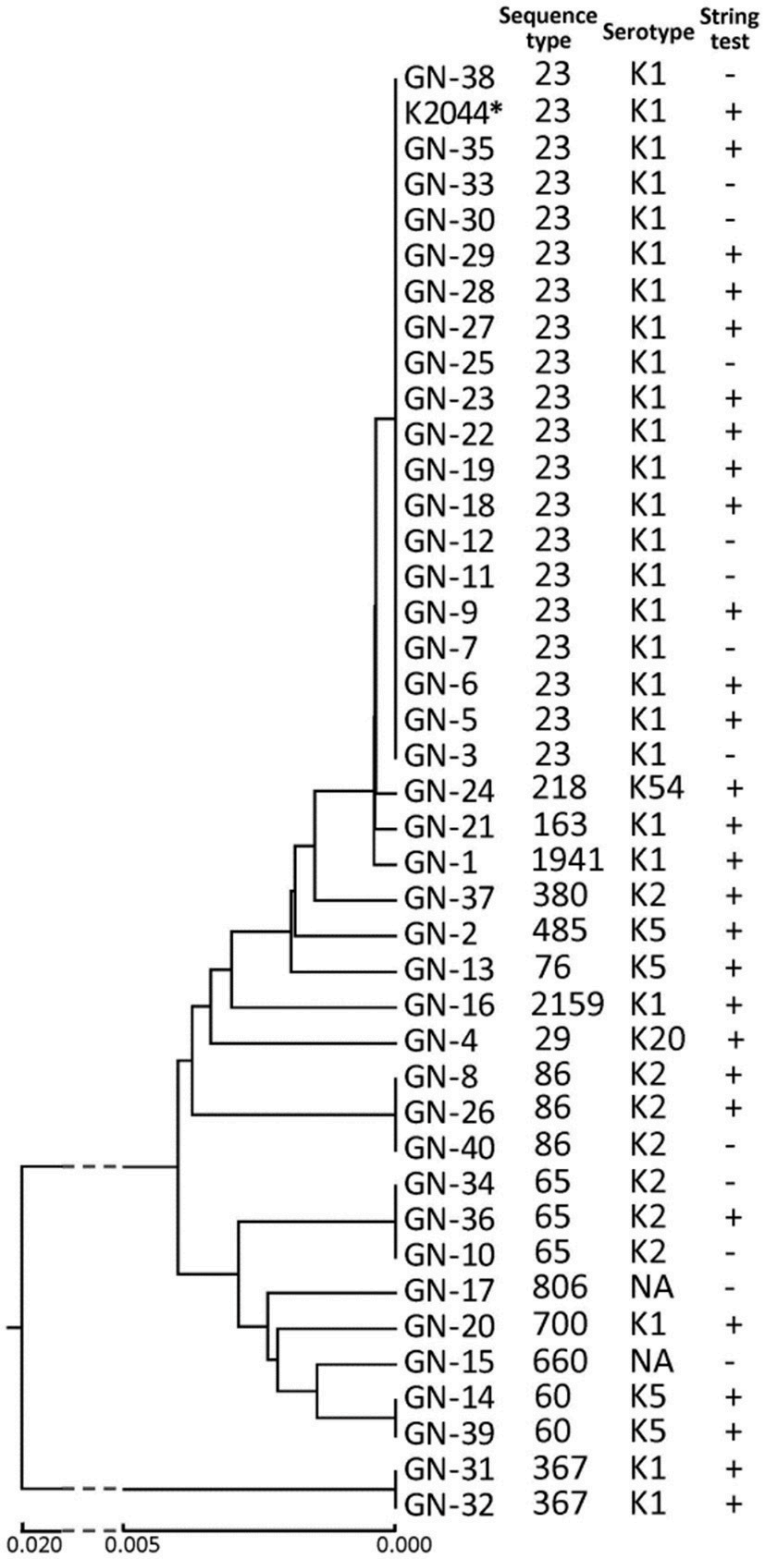
**Microbiological characteristics of 40 *K. pneumoniae* isolates from liver abscess drainage**. Phylogenetic tree was derived from MLST analysis of 7 housekeeping genes of *K. pneumoniae*. *K. pneumoniae* NTUH-K2044 was included in the analysis and is marked by an asterisk. NT, non-typeable. Straight line below the figure represents phylogenetic distance among isolates.

As shown in Figure 1, MLST analysis revealed a total of 16 sequence types. ST23 (*n* = 19, 47.5%) was the major sequence type. ST86 (*n* = 3) and ST65 (*n* = 3) were also identified. Six STs, ST218, ST1941, ST76, ST2159, ST660, and ST485, that have not been reported previously in LA-Kp were identified. K1 serotype was the dominant serotype (62.5%), followed by K2 (17.5%) and K5 (10%) (Figure 1). A majority of K1 strains (76.0%) belonged to ST23 sequence type, whereas most of K2 strains fell into ST86 and ST65. In addition, one isolate belonged to K20 and one to K54. Moreover, 2 LA-Kp isolates were non-typeable. The positive string test rate of the LA-Kp was only 67.5% (Figure 1).

### Genome sequencing and analysis of hvKp associated genes on chromosome

To further investigate the molecular and genomic features of LA-Kp, whole genome sequencing and analysis were conducted for 2 LA-Kp isolates, GN-2 and GN-3, and one multi-drug resistance (MDR) cKp isolated from blood for comparison, XL-1. GN-3 is a typical hvKp strain, which is ST23 sequence type and K1 serotype, similar to the well-studied LA-Kp strain NTUH-K2044. GN-2 (ST485, K5) was selected because over 35% LA-Kp isolates in our study are non-typical, and ST485 sequence type is a new hvKp sequence type that has not been reported to-date.

**Figure 2:**
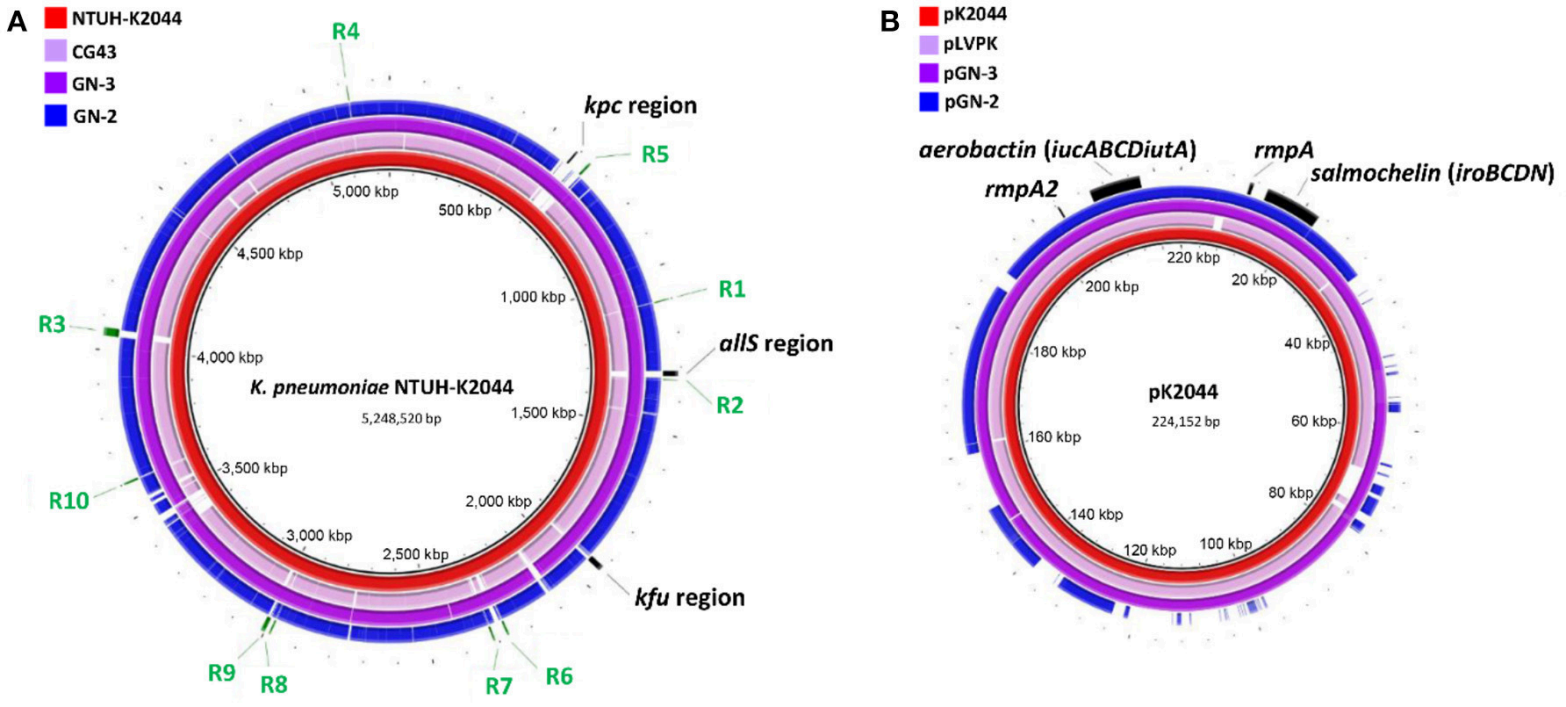
**Genomic sequence comparative analysis of 4 LA-Kp strains. (A)** Chromosome sequences comparative analysis. The chromosome sequence of *K. pneumoniae* NTUH-K2044 was used as reference for alignment. **(B)** Plasmid sequences comparative analysis. Sequence of pK2044 served as a reference sequence for comparison. All the sequences were aligned to the reference sequence by BRIG v0.95 with default parameters.

About 1.9, 1.6, and 1.7 M pairs of 300 bp pair-end reads were generated and assembled into 54, 52, and 83 contigs with final genome sizes of 5.61, 5.74, and 5.53 Mbp for GN-2, GN-3, and XL-1, respectively. The detailed information of sequenced genomes is listed in Table 3. Previously, several genes or clusters on chromosome have been reported to be associated with hvKp. We mapped these genes/clusters to the genomes of GN-2 and GN-3, as well as to other two LA causing *K. pneumoniae*, NTUH-K2044 and CG43, whose complete genomic sequences are available. *kfu* region, *allS* region and a 56-kb putative pathogenicity island *kpc*, were reported to be associated with virulence in NTUH-K2044 (Chou et al., 2004; Ma et al., 2005; Wu et al., 2010). These three regions were all found in GN-3, but not detected in GN-2 and CG43 (Figure 2A). Additional 10 genomic regions (R1–R10 in Figure 2A) consisting of 72 genes were previously reported to be associated to CC23 (Struve et al., 2015), a group of hvKp strains with most of which are ST23 sequence type. In our analysis, these 72 genes were all present in GN-3 and NTUH-K2044 (both belong to ST23, K1). However, only 3 of these genes were detected in GN-2 (ST485, K5) and CG43 (ST86, K2), including 2 genes encoding for CRISPR-associated proteins and one encoding for a hypothetical protein.

**Table 3:** **General genome information of six *K. pneumoniae* strains used in this study**.

**Characteristics^#^**	**LA-Kp**			**Non-LA-Kp**		
	**GN-3**	**GN-2**	**NTUH-K2044**	**XL-1**	**HS11286**	**MGH78578**
Genome size (Mbp)	5.74	5.61	5.47	5.53	5.68	5.69
GC content (%)	56.98	57.12	57.37	57.19	57.12	57.15
Contigs	52	54	2	83	7	6
CDS	5648	5522	5275	5463	5725	5575
CDS average length	906	904	917	899	878	901
ST^*^	ST23	ST485	ST23	ST11	ST11	ST38
Genes clustered by OrthoMCL	5394	5158	5212	5297	5313	5223
OrthoMCL clustering ratio (%)	95.50	93.41	98.81	96.96	92.80	93.69
Genes clustered by COG	4648	4587	4497	4516	4620	4605
COG clustering ratio (%)	82.29	83.07	85.25	82.67	80.70	80.81

# *Sequences of chromosome and plasmid(s) are analyzed together. Genome sequences of NTUH-K2044, HS11286, MGH78578 were published by others previously*.

* *Sequence types are assigned by online tools (http://bigsdb.web.pasteur.fr/klebsiella/klebsiella.html)*.

### LA-Kp isolates harbor plasmid(s) varied in size and number

Sequence analysis showed that both GN-2 and GN-3 harbored a plasmid (designated as pGN-2 and pGN-3 respectively) (Wu et al., 2009). pGN-2 and pGN-3 were then further compared to pK2044 and pLVPK. As shown in Figure 2B, aerobactin (*iucABCDiutA*), salmochelin (*iroBCDN*), *rmpA*, and *rmpA2*, previously reported to be associated with LA-Kp, were all detected on pGN-2 and pGN-3. pGN-3 (purple) aligned well with pK2044 (red) and pLVPK (pink). However, the sequence of pGN-2 (blue) showed nearly 40% difference from the other three plasmids. The finding of pGN-2 with variable sequences implies that not all the genes on the pK2044 and pK2044-like virulence plasmids, but a portion of the genes, are required for the hypervirluent phenotype of LA-Kp.

**Figure 3:**
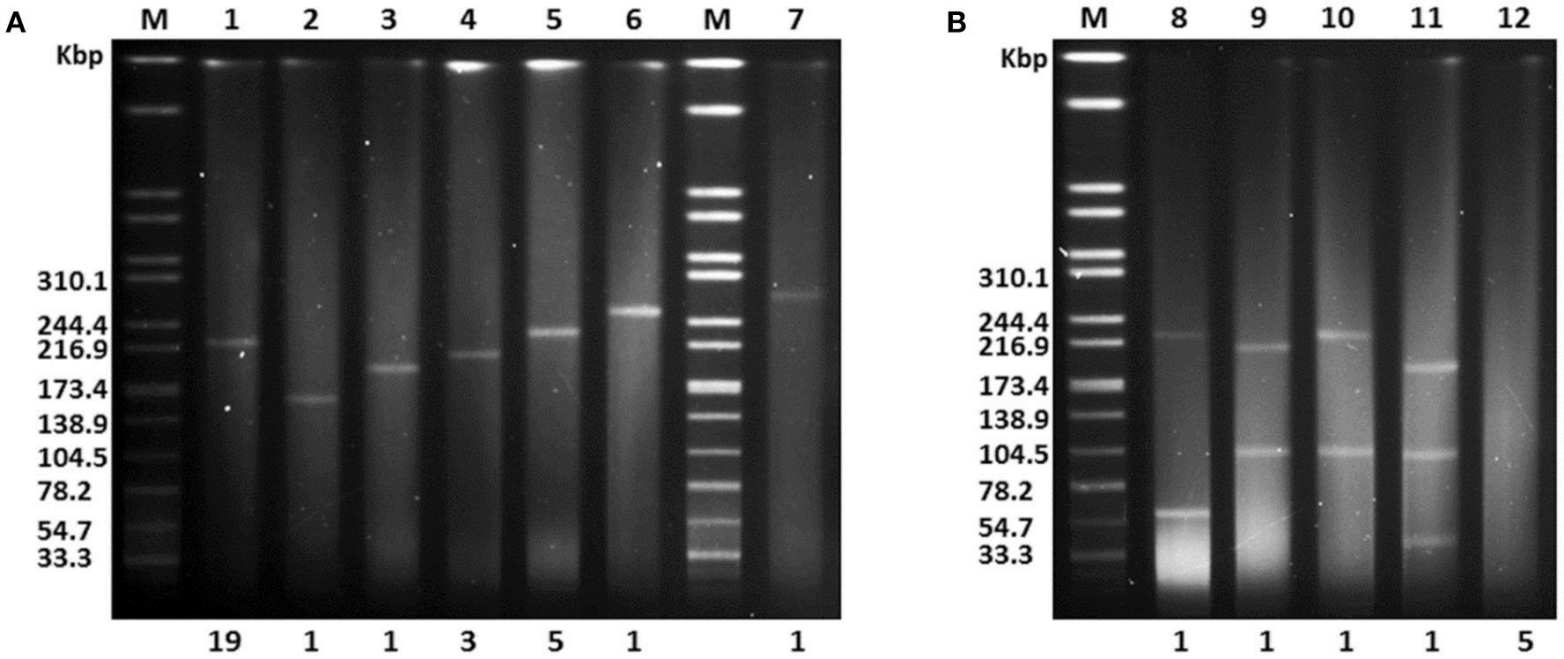
**S1-PFGE analysis of endogenous plasmid profiles for 40 LA-Kp isolates. (A)** LA-Kp that contains a single plasmid with variation in size. M, DNA marker. Numbers labeled on top are Lane number. Numbers labeled at the bottom are the number of LA-Kp isolates having the corresponding plasmid profile. **(B)** LA-Kp that contains no or multiple plasmids.

It is generally considered that hvKp strains only harbor a pK2044-like plasmid. However, limited experimental data are available supporting this hypothesis. Thus, we examined the plasmid profiles of 40 LA-Kp clinical isolates by S1-PFGE analysis. As shown in Figure 3, 12 different plasmid profiles were observed among 40 LA-Kp strains. Thirty-one strains harbored a single plasmid with different size (Figure 3A). Among them, 19 strains (61.3%, including GN-1, 3, 6, 8, 15 16, 19, 21, 22, 25, 26, 27, 28, 30, 33, 34, 36, 38, 40) contained a plasmid with similar size as pK2044 (around 220 kbp), and 12 strains (GN-37, 18, 4, 24, 32, 9, 10, 11, 20, 29, 2, 31) harbored a plasmid ranging from 140 to 250 kbp. Interestingly, 4 strains (GN-7, 23, 12, 17) harbored two or three plasmids (Figure 3B). Moreover, 5 LA-Kp strains (GN-5, 13, 14, 35, 39) contained no plasmid (Figure 3B), implying that the presence of a replicating plasmid is not essential for causing LA. Further PCR analysis showed that aerobactin/salmochelin and *rmpA*/*A2*, were all present within all 40 LA-Kp strains, including the 5 isolates with no plasmid (see below) suggesting that these “plasmid-encoded” virulence genes have been integrated into the chromosome.

### Functional comparative analyses revealed differences between LA-Kp and non-LA-Kp

**Figure 4:**
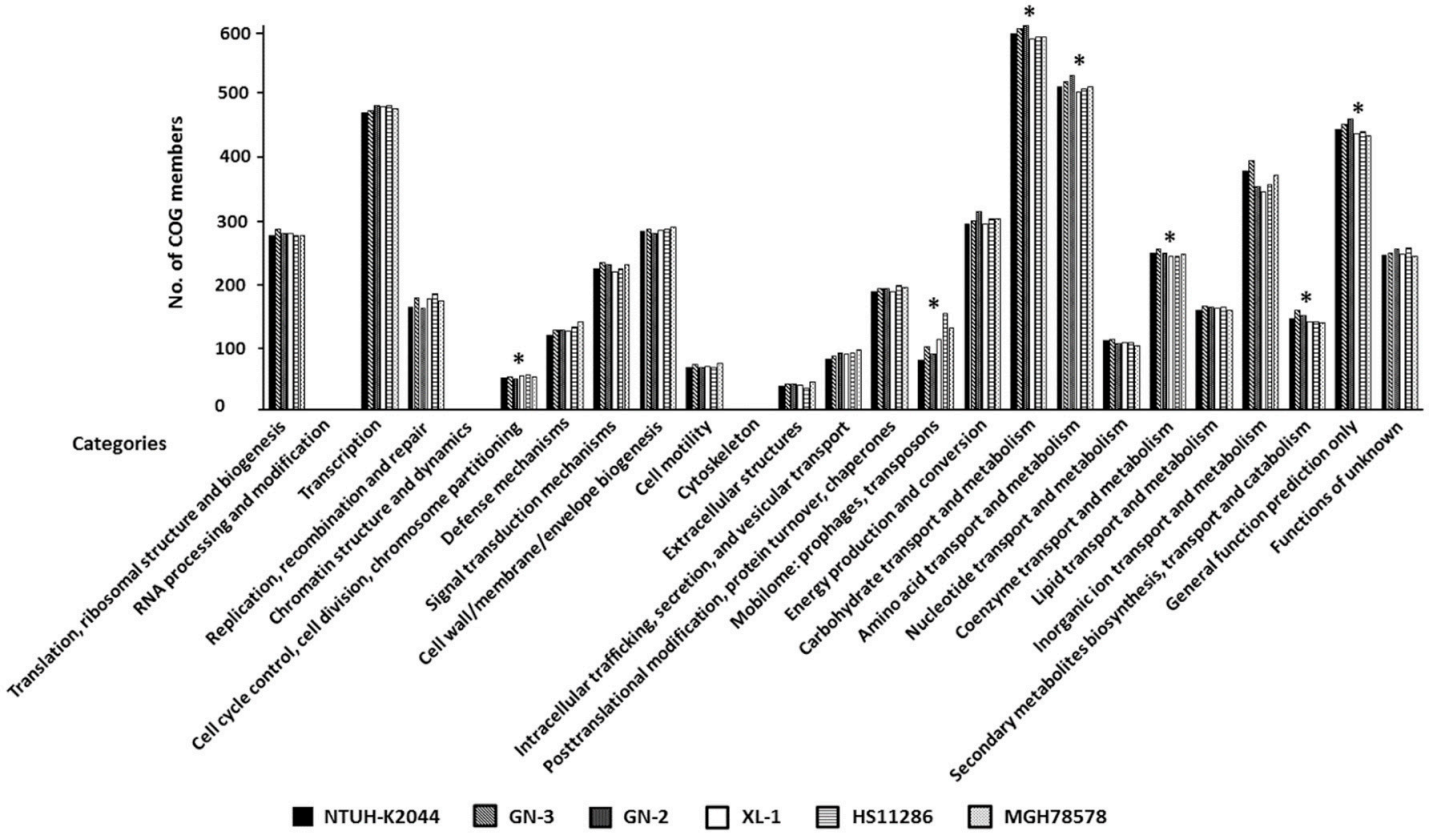
**Clusters of Orthologous Groups (COG) analysis between LA-Kp and non-LA-Kp**. Categories that were significantly different in gene numbers between LA-Kp (NTUH-K2044, GN-3, GN-2) and non-LA-Kp group (XL-1, HS11286, MGH78578) are marked with asterisk.

Functional analysis was conducted by clustering annotated genes according to 25 Clusters of Orthologous Groups (COG) categories; more than 80% of genes were clustered with COGs. As shown in Figure 4, seven COG categories showed significant difference between LA-Kp and non-LA-Kp isolates. Numbers of genes in categories of “mobilome: prophages, transposons” and “cell cycle control, cell division, chromosome partitioning” in LA-Kp were significantly less than those in non-LA-Kp. In addition, LA-Kp isolates had significantly more members of genes in other 5 categories than non-LA-Kp strains, including “carbohydrate transport and metabolism,” “amino acid transport and metabolism,” “coenzyme transport and metabolism,” “secondary metabolites biosynthesis, transport and catabolism,” and “general function prediction only.”

We also compared the distribution of antimicrobial resistance (AMR) genes between 3 LA-Kp and 3 non-LA-Kp. AMR comparative analysis showed that LA-Kp only carried one antibiotic resistance gene on chromosome (Table S3), encoding for SHV beta-lactamase, which is responsible for the resistance to ampicillin (except that GN-2 carried an additional fosfomycin resistance gene *fosA*). Three non-LA-Kp isolates carried several plasmid-encoded drug resistance genes, such as *bla*_KPC-2_, *bla*_TEM-1*B*,_ and *bla*_CTX-*M*-14_, other than the chromosomal SHV beta-lactamase gene (Table S3).

### Whole genome orthologous gene comparative analysis revealed new LA-associated virulence genes

We firstly identified the virulence factors annotated by PATRIC_VF database and compared them between 3 LA-Kp strains (GN-2, GN-3, NTUH-K2044) and 3 non-LA-Kp strains (XL-1, HS11286, and MGH78578) (Table 3). Comparative analysis identified 10 virulence genes that were uniquely present in LA-Kp but not in non-LA-Kp, including the known virulence factors aerobactin (*iucABCDiutA*) and salmochelin (*iroBCDN*), and *pagO*, a gene that has not been shown to be associated with LA previously (Table S4).

Since virulence factors recorded in the existing database are limited, orthologous gene comparative analysis of whole genome was conducted for 3 LA-Kp and 3 non-LA-Kp isolates (Table 3). Of 4649 orthologous genes shared by 3 LA-Kp isolates, 133 genes were found only present in LA-Kp genomes (Table S5). Strikingly, 84.9% of these genes (113 of 133) were located on the large virulence plasmid. To further examine the specificity of the 133 LA-associated genes identified, these genes were subjected to screening in 148 *K. pneumoniae* genome sequences obtained from public databases, including 45 isolated from LA, 44 isolated from bloodstream, and 59 from sputum and urine (general information of these strains was listed in Table S2). A gene with an occurrence frequency in LA-Kp more than 75% was considered as a high prevalence gene. Of the 133 genes, 129 were highly prevalent in LA-Kp isolates. However, among the 129 genes, 63 genes were present in isolates from blood with an occurrence frequency more than 25% (Table S6), indicating that not all the 133 genes are specific to LA-Kp.

**Figure 5:**
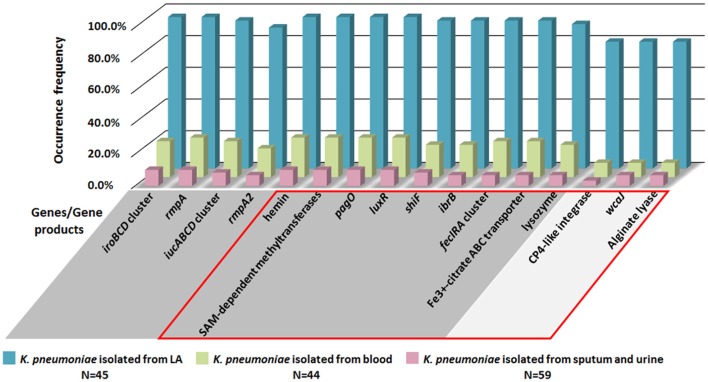
**Distributions of LA-associated genes in 148 *K. pneumoniae* isolates from public database**. Genes/gene products in GRAY background area are located on the large virulence plasmid, and genes in SILVER background area are located on chromosome. Genes/gene products within the RED box are the newly identified LA-associated genes.

To identify the genes specific to LA-Kp, we applied the accuracy rate as an indicator. Accuracy rate was calculated by dividing the sum of true positive cases in LA-Kp group and true negative cases in non-LA-Kp group with the total number of cases examined. A gene with an accuracy rate greater than 85% was designated as a highly associated gene. Upon calculating the accuracy rate for 45 LA-Kp and 44 *K. pneumoniae* isolates from blood, 30 out of 133 genes were identified as highly LA-associated genes (Table 4). Of the 30 highly associated genes, 9 were *iroBCD, iucABCD*, and *rmpA*/*A2*. In addition to *pagO* identified above, 20 newly identified LA-associated genes shared highly similar distribution with *iroBCD, iucABCD*, and *rmpA*/*A2* in LA-Kp and non-LA-Kp from blood (Figure 5, genes with unknown function were not shown). Among the 30 genes, 27 are located on the plasmid, whereas 3 genes are on the chromosome (Table 4). Furthermore, these 30 genes showed low prevalence (6–10%) among 59 genome sequences of *K. pneumoniae* isolates from sputum and urine (which are generally considered as cKp) (Figure 5). The accuracy rates of these genes were high (88–94%) in distinguishing LA-Kp from *K. pneumoniae* isolated from sputum and urine (Table S7). Thus, these newly identified 21 genes are highly associated with LA-Kp.

**Table 4:** **30 LA-associated genes identified in this study**.

Genebank ID^*^	**Location^#^**	**Common name**
BAH65933.1	P	ABC transporter permease protein, hemin
BAH65942.1	P	Mobile element protein
BAH65943.1	P	Putative SAM-dependent methyltransferases
BAH65944.1	P	Regulator of mucoid phenotype A, RmpA
BAH65945.1	P	Conserved hypothetical protein
BAH65946.1	P	DNA-binding response regulator, LuxR
BAH65947.1	P	Metabolite transporter (DMT) superfamily, PagO
BAH65948.1	P	Conserved hypothetical protein
BAH65950.1	P	Trilactone hydrolase, IroD
BAH65951.1	P	ABC transporter protein, IroC
BAH65952.1	P	Glycosyltransferase, IroB
BAH65954.1	P	Co-activator of prophage gene expression, IbrB
BAH65956.1	P	Mobile element protein
BAH65957.1	P	Mobile element protein
BAH65959.1	P	RNA polymerase sigma factor, FecI
BAH(26153..26674)	P	Iron(III) dicitrate transmembrane sensor protein, FecR
BAH(27079..26957)	P	Conserved hypothetical protein
BAH65960.1	P	Iron(III) dicitrate transport protein, FecA
BAH65961.1	P	Fe^3+^-citrate ABC transporter
BAH65944.1	P	Regulator of mucoid phenotype A2, RmpA2
BAH66194.1	P	Aerobactin biosynthesis protein, IucD
BAH66195.1	P	Aerobactin biosynthesis protein, IucC
BAH66196.1	P	Aerobactin biosynthesis protein, IucB
BAH66197.1	P	Aerobactin biosynthesis protein, IucA
BAH66198.1	P	Possible H+-antiporter clustered with aerobactin genes, ShiF
BAH66199.1	P	Conserved hypothetical protein
BAH66205.1	P	Periplasmic lysozyme inhibitor of c-type lysozyme
BAH61925.1	C	Site-specific recombinase, phage integrase family, CP4-like integrase
BAH64287.1	C	Colanic acid biosynthsis UDP-glucose lipid carrier transferase, WcaJ
BAH64342.1	C	Alginate lyase

* *Genebank ID of NTUH-K2044 recorded in NCBI; Genes that were not annotated in NCBI were presented as BAH(start position.end position)*.

# *P represents genes located on plasmid; C represents genes located on chromosome*.

Among the 21 predicted LA-associated genes (Table 4), *pagO* encodes for a PhoPQ-activated integral membrane protein and is required for virulence in *Salmonellae* spp. (Gunn et al., 1998). Genetic screening suggests that *pagO* in LA-Kp may be required for liver abscess induction in a mouse model (Tu et al., 2009). SAM-dependent methyltransferase has been reported to play an important role in the adaptation to hostile environment of the macrophage and acid stress in *Mycobacterium tuberculosis* (Healy et al., 2016). *shiF* has been speculated as an auxiliary gene that promotes the transport of lysine, the precursor of aerobactin (*iucABCD*) in *E. coli*. Its expression is upregulated when exposed to chicken serum or iron-deficient environment (Lemaitre et al., 2012). In *Klebsiella pneumoniae, shiF* is located adjacent to *iucABCD* which further supports its potential role in virulence. LuxR is a key quorum-sensing regulator in controlling the virulence gene expression in many bacteria, such as *Vibrio alginolyticus* and *Xanthomonas oryzae* (Xu et al., 2015; Gu et al., 2016). Several other genes including hemin and *fecIRA* gene cluster are associated with iron acquisition, suggesting that they may play a key role in hvKp pathogenicity similar to aerobactin and salmochelin. Other genes were mobile elements and phage-related genes, which may contribute to the integration of plasmid into chromosome.

### Distribution of newly identified LA-associated virulence genes among clinical *K. pneumoniae* isolates

**Figure 6:**
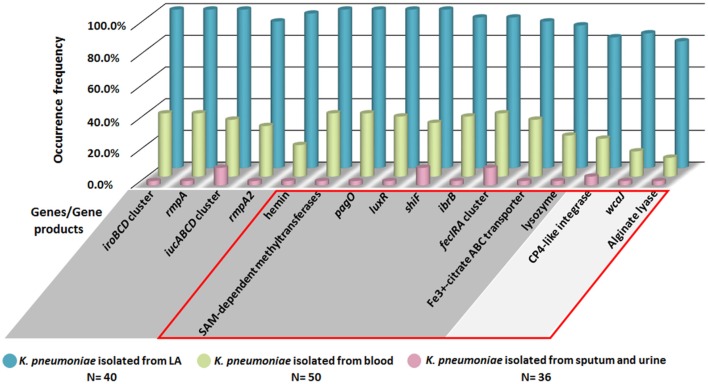
**PCR analysis of the distribution of LA-associated genes among 126 clinical *K. pneumoniae* isolates**. Genes/gene products in GRAY background area are located on the large virulence plasmid, and genes in SILVER background area are located on chromosome. Genes/gene products within the RED box are the newly identified LA-associated genes.

To confirm the specificity of LA-associated virulence genes identified by bioinformatics analysis, PCR analysis was performed for a totally 126 clinical *K. pneumoniae* isolates, including 40 isolates from liver abscess, 50 from blood and 36 from sputum or urine in patients without bloodstream infection. As shown in Figure 6, in addition to *iroBCD, iucABCD* and *rmpA/A2, four* newly identified LA-associated genes including the gene encoding SAM-dependent methyltransferases, *pagO, luxR*, and *shiF* were 100% present in LA-Kp, and only 2–11% in *K. pneumoniae* isolates from sputum or urine. The occurrences of other newly predicted LA-associated genes including *ibrB, fecIRA* cluster, *wcaJ*, and genes encoding Fe^3+^-citrate ABC transporter, lysozyme, CP4-like integrase, and alginate lyase, were also significantly higher in *K. pneumoniae* isolated from liver abscess (80–97%) than those in *K. pneumoniae* isolates from sputum or urine (2–11%).

## Discussion

We have just begun to understand the nature of hvKp, and many questions regarding the hypervirulent nature of this pathogen remain to be answered. In this study, we isolated 40 LA-Kp strains from most recent PLA cases in mainland China. We found that over 90% of pathogens isolated from surgical drainage of PLA were *K. pneumoniae*. The majority of patients had underlying disease of diabetes. The vast majority of the LA-Kp were susceptible to main antimicrobial agents. K1/K2 serotype contributed to most of LA-Kp and ST23 was the dominant sequence type.

Sequencing and S1-PFGE analyses in this study led to one of the important findings that there is a diverse plasmid profile among hvKp strains. It has been thought that all hvKp strains harbor a pK2044-like plasmid that carries genes encoding siderophores (aerobactin/salmochelin) and *rmpA/A2* which are critical for the virulence of LA-Kp (Struve et al., 2015). We first identified a plasmid pGN2 in hvKp that is dramatically different from pK2044. We then examined the plasmid profiles of all 40 LA-Kp clinical isolates by S1-PFGE and found that 77.5% of the LA-Kp isolates harbor a single pK2044-like large virulence plasmid, and 12.5% isolates have no plasmid. In addition, 10% of LA-Kp isolates contained two or three plasmids. To the best of our knowledge, this phenomenon has not been reported in LA-Kp isolates or any other hvKp strains heretofore. This is important since it has been postulated that hvKp strains are resistant to acquire plasmids, which may account for the observation that hvKp tend to be highly antimicrobial susceptible (Alcántar-Curiel and Giron, 2015). Our finding that the coexistence of the large virulent plasmid and other plasmids suggests that hvKp could acquire other plasmids. A recent study reported that the *bla*_CTX-*M*_ carrying plasmids could be acquired by hvKP (Zhang et al., 2016). Therefore, a concern of multidrug-resistant hvKP infection might be raised.

For the 5 LA-Kp isolates that contain no plasmid, we hypothesized that the plasmid has integrated into the chromosome based on the fact that these isolates all have aerobactin, salmochelin and *rmpA/A2*. Tang et al. also reported several K1 and K54 plasmidless hvKp isolates in Taiwan, and demonstrated that pLVPK (a plasmid similar to pK2044) was integrated into the chromosome (designated as chromosome-integrated form) in these isolates (Tang et al., 2010). Among our chromosome-integrated isolates, 3 belonged to K5 serotypes and 2 belonged to K1. Given that a large plasmid is likely difficult to be maintained by the cell, integration of virulent plasmid into the chromosome may provide an advantage for hvKp to maintain the virulence factors and its invasive nature.

Besides aerobactin, salmochelin and *rmpA/A2*, little is known for other virulence genes in hvKp. Previous studies suggested that the chromosomally carried *allS* gene cluster, *kfu* gene cluster and *kpc* fimbrial gene cluster were associated to virulence in hvKp K1 serotype strains (Chou et al., 2004; Ma et al., 2005; Wu et al., 2010). Recent genome comparative study focusing on *K. pneumoniae* CC23 isolates suggests that these clusters were mainly associated to CC23 or K1 serotype. CC23 or K1 serotype strains also include some non-hvKp (Liu et al., 2014). In our analysis, clusters of *alls, kfu, kpc* fimbrial and 10 genomic regions were not detected in GN-2 (ST485, K5) and *K. pneumoniae* CG43 (ST86, K2). Thus, these clusters may contribute to virulence in CC23 or K1 serotype isolates, or may be the conserved genes but not virulence-associated.

In our study, over 40% LA-Kp clinical isolates are non-CC23. Therefore, we performed genomic comparative analysis between groups of LA-Kp (including non-CC23) and non-LA-Kp for identification of the virulence factors in LA-Kp. First, we compared the known virulence genes in PATRIC virulence database between 3 LA-Kp and 3 non-LA-Kp and identified a new LA-Kp associated virulence factor, *pagO*. Considering that the virulence genes in database are limited, we then compared all the genes in 3 LA-Kp strains to genes in 3 non-LA-Kp strains based on their orthologous relationships, and identified 133 genes that were present only in LA-Kp, including *iucABCD, iroBCD and pagO*. We further tested the specificity of the 133 genes to LA-Kp by their prevalence in 45 LA-Kp and 103 non-LA-Kp genome sequences, which led to identification of 21 new genes that are highly associated with LA-Kp. Finally, we tested the prevalence of the newly predicted genes in 40 LA-Kp and 86 non-LA-Kp clinical isolates by PCR analysis. The result showed that the genes encoding SAM-dependent methyltransferases, *pagO, luxR*, and *shiF* were present 100% in LA-Kp, but only 2–11% in isolates from sputum or urine. Other genes tested showed 80–97% in LA-Kp and only 2–11% in isolates from sputum or urine. Thus, the specificity test results of these genes in clinical isolates supports bioinformatics prediction.

Several of the 21 newly predicted LA-associated genes identified in this study have been shown to be involved in virulence in other bacterial species, supporting the hypothesis that these genes are potential virulence genes in LA-Kp. A genetic screening study showed that one of these genes, *pagO*, has been shown to be involved in liver abscess formation by LA-Kp (Tu et al., 2009). In that study, an oral infection model was established for liver abscess formation by LA-Kp. Using a signature-tagged transposon mutant library, they identified 28 genes whose mutations resulted in reduced ability to develop liver abscess. However, no complementation experiment was performed to confirm the mutant phenotype. One caveat of our study is that roles of those predicted virulence factors in LA formation have not been tested genetically. We are currently in the process of performing genetics to elucidate the functions of those genes in LA-Kp virulence.

## Author contributions

MY, JT, MW, and JH designed the study. MY and JT drafted the manuscript. JT, YZ, ZC, ZY, CS, JY, and XZ collected clinical isolates and clinical data. JJ conducted the bioinformatic analyses. MY, YB, WY, JR, TZ, ZS, and BD carried out experiments. QG and XX raised several useful suggestions. All authors read and approved the final manuscript.

### Conflict of interest statement

The authors declare that the research was conducted in the absence of any commercial or financial relationships that could be construed as a potential conflict of interest.

## Abbreviations

LA: liver abscess
PLA: pyogenic liver abscess
KLA: *Klebsiella pneumoniae*-caused pyogenic liver abscess
LA-Kp: liver abscess causing *K. pneumoniae*
hvKp: hypervirulent variants of *K. pneumoniae*
cKp: “classic” *K. pneumoniae*.

## References

[B1] Alcántar-Curiel M. D. Girón J. A. (2015). *Klebsiella pneumoniae* and the pyogenic liver abscess: implications and association of the presence of rpmA genes and expression of hypermucoviscosity. Virulence 6, 407–409. 10.1080/21505594.2015.1030101 25951089 PMC4601161

[B2] Alikhan N. F. Petty N. K. Ben Zakour N. L. Beatson S. A. (2011). BLAST Ring Image Generator (BRIG): simple prokaryote genome comparisons. BMC Genomics 12:402. 10.1186/1471-2164-12-402 21824423 PMC3163573

[B3] Bankevich A. Nurk S. Antipov D. Gurevich A. A. Dvorkin M. Kulikov A. S.. (2012). SPAdes: a new genome assembly algorithm and its applications to single-cell sequencing. J. Comput. Biol. 19, 455–477. 10.1089/cmb.2012.0021 22506599 PMC3342519

[B4] Barton B. M. Harding G. P. Zuccarelli A. J. (1995). A general method for detecting and sizing large plasmids. Anal. Biochem. 226, 235–240. 10.1006/abio.1995.1220 7793624

[B5] Bialek-Davenet S. Criscuolo A. Ailloud F. Passet V. Jones L. Delannoy-Vieillard A. S.. (2014). Genomic definition of hypervirulent and multidrug-resistant *Klebsiella pneumoniae* clonal groups. Emerging Infect. Dis. 20, 1812–1820. 10.3201/eid2011.140206 25341126 PMC4214299

[B6] Brisse S. Fevre C. Passet V. Issenhuth-Jeanjean S. Tournebize R. Diancourt L.. (2009). Virulent Clones of *Klebsiella pneumoniae*: identification and evolutionary scenario based on genomic and phenotypic characterization. PLoS ONE 4:e4982. 10.1371/journal.pone.0004982 19319196 PMC2656620

[B7] Chen Y. T. Chang H. Y. Lai Y. C. Pan C. C. Tsai S. F. Peng H. L. (2004). Sequencing and analysis of the large virulence plasmid pLVPK of *Klebsiella pneumoniae* CG43. Gene 337, 189–198. 10.1016/j.gene.2004.05.008 15276215

[B8] Cheng D. L. Lin C. L. (1986). *Klebsiella pneumoniae* liver abscess associated with septic endophthalmitis. Arch. Intern. Med. 146, 1913–1916. 3532983

[B9] Chou H. C. Lee C. Z. Ma L. C. Fang C. T. Chang S. C. Wang J. T. (2004). Isolation of a chromosomal region of *Klebsiella pneumoniae* associated with allantoin metabolism and liver infection. Infect. Immun. 72, 3783–3792. 10.1128/IAI.72.7.3783-3792.2004 15213119 PMC427404

[B10] Chung D. R. Lee H. R. Lee S. S. Kim S. W. Chang H. H. Jung S. I.. (2008). Evidence for clonal dissemination of the serotype K1 *Klebsiella pneumoniae* strain causing invasive liver abscesses in Korea. J. Clin. Microbiol. 46, 4061–4063. 10.1128/jcm.01577-08 18971367 PMC2593285

[B11] Etienne K. A. Gillece J. Hilsabeck R. Schupp J. M. Colman R. Lockhart S. R.. (2012). Whole genome sequence typing to investigate the Apophysomyces outbreak following a tornado in Joplin, Missouri, 2011. PLoS ONE 7:e49989. 10.1371/journal.pone.0049989 23209631 PMC3507928

[B12] Galperin M. Y. Makarova K. S. Wolf Y. I. Koonin E. V. (2015). Expanded microbial genome coverage and improved protein family annotation in the COG database. Nucleic Acids Res 43, D261–D269. 10.1093/nar/gku1223 25428365 PMC4383993

[B13] Gu D. Guo M. Yang M. Zhang Y. Zhou X. Wang Q. (2016). A sigmaE-mediated temperature gauge controls a switch from LuxR-mediated virulence gene expression to thermal stress adaptation in *Vibrio alginolyticus*. PLoS Pathog. 12:e1005645. 10.1371/journal.ppat.1005645 27253371 PMC4890791

[B14] Gunn J. S. Belden W. J. Miller S. I. (1998). Identification of PhoP-PhoQ activated genes within a duplicated region of the *Salmonella typhimurium* chromosome. Microb. Pathog. 25, 77–90. 10.1006/mpat.1998.0217 9712687

[B15] Healy C. Golby P. MacHugh D. E. Gordon S. V. (2016). The MarR family transcription factor Rv1404 coordinates adaptation of *Mycobacterium tuberculosis* to acid stress via controlled expression of Rv1405c, a virulence-associated methyltransferase. Tuberculosis (Edinb.) 97, 154–162. 10.1016/j.tube.2015.10.003 26615221

[B16] Holt K. E. Wertheim H. Zadoks R. N. Baker S. Whitehouse C. A. Dance D.. (2015). Genomic analysis of diversity, population structure, virulence, and antimicrobial resistance in *Klebsiella pneumoniae*, an urgent threat to public health. Proc. Natl. Acad. Sci. U.S.A. 112, E3574–E3581. 10.1073/pnas.1501049112 26100894 PMC4500264

[B17] Hsieh P. F. Lin T. L. Lee C. Z. Tsai S. F. Wang J. T. (2008). Serum-induced iron-acquisition systems and TonB contribute to virulence in *Klebsiella pneumoniae* causing primary pyogenic liver abscess. J. Infect. Dis. 197, 1717–1727. 10.1086/588383 18433330

[B18] Hsu C. R. Lin T. L. Chen Y. C. Chou H. C. Wang J. T. (2011). The role of *Klebsiella pneumoniae* rmpA in capsular polysaccharide synthesis and virulence revisited. Microbiology 157(Pt 12), 3446–3457. 10.1099/mic.0.050336-0 21964731

[B19] Hu B. S. Lau Y. J. Shi Z. Y. Lin Y. H. (1999). Necrotizing fasciitis associated with *Klebsiella pneumoniae* liver abscess. Clin. Infect. Dis. 29, 1360–1361. 10.1086/313471 10525011

[B20] Hyun J. I. Kim Y. J. Jeon Y. H. Kim S. I. Park Y. J. Kang M. W.. (2014). A case of ventriculitis associated with renal abscess caused by Serotype K1 *Klebsiella pneumoniae*. Infect Chemother 46, 120–124. 10.3947/ic.2014.46.2.120 25024876 PMC4091369

[B21] Inouye M. Dashnow H. Raven L. A. Schultz M. B. Pope B. J. Tomita T.. (2014). SRST2: rapid genomic surveillance for public health and hospital microbiology labs. Genome Med. 6:90. 10.1186/s13073-014-0090-6 25422674 PMC4237778

[B22] Kim J. H. Yang W. J. Kim T. H. (2014). *Klebsiella pneumonia*-induced prostate abscess: how to work it up? Can. Urol. Assoc. J. 8, E841–E844. 10.5489/cuaj.2155 25485013 PMC4250250

[B23] Lemaitre C. Bidet P. Bingen E. Bonacorsi S. (2012). Transcriptional analysis of the *Escherichia coli* ColV-Ia plasmid pS88 during growth in human serum and urine. BMC Microbiol. 12:115. 10.1186/1471-2180-12-115 22720670 PMC3438092

[B24] Li L. S. He N. S. (2001). Interventional Radiology-Non Vascular. Beijing: People's Medical Publishing House.

[B25] Li L. Stoeckert C. J. Jr. Roos D. S. (2003). OrthoMCL: identification of ortholog groups for eukaryotic genomes. Genome Res. 13, 2178–2189. 10.1101/gr.1224503 12952885 PMC403725

[B26] Lin J. C. Chang F. Y. Fung C. P. Xu J. Z. Cheng H. P. Wang J. J.. (2004). High prevalence of phagocytic-resistant capsular serotypes of *Klebsiella pneumoniae* in liver abscess. Microbes Infect. 6, 1191–1198. 10.1016/j.micinf.2004.06.003 15488738

[B27] Lin J. C. Koh T. H. Lee N. Fung C. P. Chang F. Y. Tsai Y. K.. (2014). Genotypes and virulence in serotype K2 *Klebsiella pneumoniae* from liver abscess and non-infectious carriers in Hong Kong, Singapore and Taiwan. Gut Pathog. 6:21. 10.1186/1757-4749-6-21 24987462 PMC4076766

[B28] Liu Y. M. Li B. B. Zhang Y. Y. Zhang W. Shen H. Li H.. (2014). Clinical and molecular characteristics of emerging hypervirulent *Klebsiella pneumoniae* bloodstream infections in mainland China. Antimicrob. Agents Chemother. 58, 5379–5385. 10.1128/aac.02523-14 24982067 PMC4135864

[B29] Luo Y. Wang Y. Ye L. Yang J. (2014). Molecular epidemiology and virulence factors of pyogenic liver abscess causing *Klebsiella pneumoniae* in China. Clin. Microbiol. Infect. 20, O818–O824. 10.1111/1469-0691.12664 24804560

[B30] Ma L. C. Fang C. T. Lee C. Z. Shun C. T. Wang J. T. (2005). Genomic heterogeneity in *Klebsiella pneumoniae* strains is associated with primary pyogenic liver abscess and metastatic infection. J. Infect. Dis. 192, 117–128. 10.1086/430619 15942901

[B31] Merlet A. Cazanave C. Dutronc H. de Barbeyrac B. Brisse S. Dupon M. (2012). Primary liver abscess due to CC23-K1 virulent clone of *Klebsiella pneumoniae* in France. Clin. Microbiol. Infect. 18, E338–E339. 10.1111/j.1469-0691.2012.03953.x 22757694

[B32] Mizuta K. Ohta M. Mori M. Hasegawa T. Nakashima I. Kato N. (1983). Virulence for mice of Klebsiella strains belonging to the O1 group: relationship to their capsular (K) types. Infect. Immun. 40, 56–61. 6187694 10.1128/iai.40.1.56-61.1983 PMC264817

[B33] Nassif X. Sansonetti P. J. (1986). Correlation of the virulence of *Klebsiella pneumoniae* K1 and K2 with the presence of a plasmid encoding aerobactin. Infect. Immun. 54, 603–608. 2946641 10.1128/iai.54.3.603-608.1986 PMC260211

[B34] Nassif X. Fournier J. M. Arondel J. Sansonetti P. J. (1989). Mucoid phenotype of *Klebsiella pneumoniae* is a plasmid-encoded virulence factor. Infect. Immun. 57, 546–552. 2643575 10.1128/iai.57.2.546-552.1989 PMC313131

[B35] Podschun R. Ullmann U. (1998). Klebsiella spp. as nosocomial pathogens: epidemiology, taxonomy, typing methods, and pathogenicity factors. Clin. Microbiol. Rev. 11, 589–603. 9767057 10.1128/cmr.11.4.589 PMC88898

[B36] Pomakova D. K. Hsiao C. B. Beanan J. M. Olson R. MacDonald U. Keynan Y.. (2012). Clinical and phenotypic differences between classic and hypervirulent *Klebsiella pneumonia*: an emerging and under-recognized pathogenic variant. Eur. J. Clin. Microbiol. Infect. Dis. 31, 981–989. 10.1007/s10096-011-1396-6 21918907

[B37] Qu T. T. Zhou J. C. Jiang Y. Shi K. R. Li B. Shen P.. (2015). Clinical and microbiological characteristics of *Klebsiella pneumoniae* liver abscess in East China. BMC Infect. Dis. 15:161. 10.1186/s12879-015-0899-7 25886859 PMC4381403

[B38] Saccente M. (1999). *Klebsiella pneumoniae* liver abscess, endophthalmitis, and meningitis in a man with newly recognized diabetes mellitus. Clin. Infect. Dis. 29, 1570–1571. 10.1086/313539 10585817

[B39] Shang H. Wang Y. S. Shen Z. Y. (2015). National Guide to Clinical Laboratory Procedures. Beijing: People's Medical Publishing House.

[B40] Shon A. S. Bajwa R. P. Russo T. A. (2013). Hypervirulent (hypermucoviscous) *Klebsiella pneumoniae*: a new and dangerous breed. Virulence 4, 107–118. 10.4161/viru.22718 23302790 PMC3654609

[B41] Siu L. K. Fung C. P. Chang F. Y. Lee N. Yeh K. M. Koh T. H.. (2011). Molecular typing and virulence analysis of serotype K1 *Klebsiella pneumoniae* strains isolated from liver abscess patients and stool samples from noninfectious subjects in Hong Kong, Singapore, and Taiwan. J. Clin. Microbiol. 49, 3761–3765. 10.1128/JCM.00977-11 21900521 PMC3209116

[B42] Siu L. K. Yeh K. M. Lin J. C. Fung C. P. Chang F. Y. (2012). *Klebsiella pneumoniae* liver abscess: a new invasive syndrome. Lancet Infect. Dis. 12, 881–887. 10.1016/S1473-3099(12)70205-0 23099082

[B43] Struve C. Roe C. C. Stegger M. Stahlhut S. G. Hansen D. S. Engelthaler D. M.. (2015). Mapping the Evolution of Hypervirulent *Klebsiella pneumoniae*. MBio 6:e00630. 10.1128/mBio.00630-15 26199326 PMC4513082

[B44] Tan T. Y. Cheng Y. Ong M. Ng L. S. (2014). Performance characteristics and clinical predictive value of the string test for detection of hepato-virulent *Klebsiella pneumoniae* isolated from blood cultures. Diagn. Microbiol. Infect. Dis. 78, 127–128. 10.1016/j.diagmicrobio.2013.10.014 24321354

[B45] Tang H. L. Chiang M. K. Liou W. J. Chen Y. T. Peng H. L. Chiou C. S.. (2010). Correlation between *Klebsiella pneumoniae* carrying pLVPK-derived loci and abscess formation. Eur. J. Clin. Microbiol. Infect. Dis. 29, 689–698. 10.1007/s10096-010-0915-1 20383552

[B46] Tu Y. C. Lu M. C. Chiang M. K. Huang S. P. Peng H. L. Chang H. Y.. (2009). Genetic requirements for *Klebsiella pneumoniae*-induced liver abscess in an oral infection model. Infect. Immun. 77, 2657–2671. 10.1128/IAI.01523-08 19433545 PMC2708586

[B47] Turton J. F. Perry C. Elgohari S. Hampton C. V. (2010). PCR characterization and typing of *Klebsiella pneumoniae* using capsular type-specific, variable number tandem repeat and virulence gene targets. J. Med. Microbiol. 59(Pt 5), 541–547. 10.1099/jmm.0.015198-0 20110386

[B48] Wattam A. R. Abraham D. Dalay O. Disz T. L. Driscoll T. Gabbard J. L.. (2014). PATRIC, the bacterial bioinformatics database and analysis resource. Nucleic Acids Res. 42, D581–D591. 10.1093/nar/gkt1099 24225323 PMC3965095

[B49] Wu C. C. Huang Y. J. Fung C. P. Peng H. L. (2010). Regulation of the *Klebsiella pneumoniae* Kpc fimbriae by the site-specific recombinase KpcI. Microbiology 156(Pt 7), 1983–1992. 10.1099/mic.0.038158-0 20378654

[B50] Wu K. M. Li L. H. Yan J. J. Tsao N. Liao T. L. Tsai H. C.. (2009). Genome sequencing and comparative analysis of *Klebsiella pneumoniae* NTUH-K2044, a strain causing liver abscess and meningitis. J. Bacteriol. 191, 4492–4501. 10.1128/JB.00315-09 19447910 PMC2704730

[B51] Xu H. Zhao Y. Qian G. Liu F. (2015). XocR, a LuxR solo required for virulence in *Xanthomonas oryzae* pv. oryzicola. Front. Cell. Infect. Microbiol. 5:37. 10.3389/fcimb.2015.00037 25932456 PMC4399327

[B52] Ye J. McGinnis S. Madden T. L. (2006). BLAST: improvements for better sequence analysis. Nucleic Acids Res. 34, W6–W9. 10.1093/nar/gkl164 16845079 PMC1538791

[B53] Yeh K. M. Lin J. C. Yin F. Y. Fung C. P. Hung H. C. Siu L. K.. (2010). Revisiting the importance of virulence determinant magA and its surrounding genes in *Klebsiella pneumoniae* causing pyogenic liver abscesses: exact role in serotype K1 capsule formation. J. Infect. Dis. 201, 1259–1267. 10.1086/606010 19785524

[B54] Yu W. L. Ko W. C. Cheng K. C. Lee C. C. Lai C. C. Chuang Y. C. (2008). Comparison of prevalence of virulence factors for *Klebsiella pneumoniae* liver abscesses between isolates with capsular K1/K2 and non-K1/K2 serotypes. Diagn. Microbiol. Infect. Dis. 62, 1–6. 10.1016/j.diagmicrobio.2008.04.007 18486404

[B55] Zankari E. Hasman H. Cosentino S. Vestergaard M. Rasmussen S. Lund O.. (2012). Identification of acquired antimicrobial resistance genes. J. Antimicrob. Chemother. 67, 2640–2644. 10.1093/jac/dks261 22782487 PMC3468078

[B56] Zhang Y. Zhao C. Wang Q. Wang X. Chen H. Li H.. (2016). High prevalence of hypervirulent *Klebsiella pneumoniae* infection in China: geographic distribution, clinical characteristics and antimicrobial resistance. Antimicrob. Agents Chemother. 60, 6115–6120. 10.1128/AAC.01127-16 27480857 PMC5038323

